# Supplementary material for: Mutations in PCYT2 disrupt etherlipid biosynthesis and cause a complex hereditary spastic paraplegia
Source: Brain. 2019 Oct 22;142(11):3382–97. doi: 10.1093/brain/awz291 (PMC6821184; doi:10.1093/brain/awz291)
Supplement: awz291_Supplementary_Data [file awz291_supplementary_data.zip › awz291-Suppl_data/Supplementary_Data4.zip!/Supplementary information 3 - fibroblast lipidomics.zip!/Fibroblast lipidomics/Controle vs Patient2/Statistical plots and data/PS species/Boxplots all.pdf]

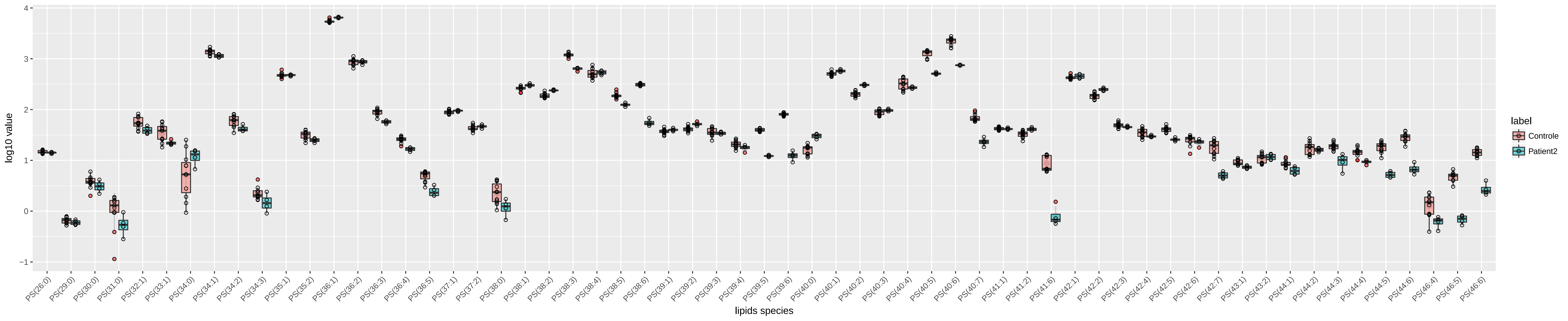

log10 value
4
3
2
1
0
−1
PS(26:0) PS(29:0) PS(30:0) PS(31:0) PS(32:1) PS(33:1) PS(34:0) PS(34:1) PS(34:2) PS(34:3) PS(35:1) PS(35:2) PS(36:1) PS(36:2) PS(36:3) PS(36:4) PS(36:5) PS(37:1) PS(37:2) PS(38:0) PS(38:1) PS(38:2) PS(38:3) PS(38:4) PS(38:5) PS(38:6) PS(39:1) PS(39:2) PS(39:3) PS(39:4) PS(39:5) PS(39:6) PS(40:0) PS(40:1) PS(40:2) PS(40:3) PS(40:4) PS(40:5) PS(40:6) PS(40:7) PS(41:1) PS(41:2) PS(41:6) PS(42:1) PS(42:2) PS(42:3) PS(42:4) PS(42:5) PS(42:6) PS(42:7) PS(43:1) PS(43:2) PS(44:1) PS(44:2) PS(44:3) PS(44:4) PS(44:5) PS(44:6) PS(46:4) PS(46:5) PS(46:6)
lipids species
label
Controle
Patient2
